# PockDrug-Server: a new web server for predicting pocket druggability on holo and apo proteins

**DOI:** 10.1093/nar/gkv462

**Published:** 2015-05-08

**Authors:** Hiba Abi Hussein, Alexandre Borrel, Colette Geneix, Michel Petitjean, Leslie Regad, Anne-Claude Camproux

**Affiliations:** 1 INSERM, UMRS-973, MTi, Université Paris Diderot, 35 Rue Hélène Brion, 75205 Paris Cedex 13, case courier 7113, Paris, France; 2 Université Paris Diderot, Sorbonne Paris Cité, UMRS-973, MTi, Paris, France; 3 Division of Pharmaceutical Chemistry, Faculty of pharmacy, University of Helsinki, Viikinkaari 9 (P.O. Box 56) FI-00014, Finland

## Abstract

Predicting protein pocket's ability to bind drug-like molecules with high affinity, i.e. druggability, is of major interest in the target identification phase of drug discovery. Therefore, pocket druggability investigations represent a key step of compound clinical progression projects. Currently computational druggability prediction models are attached to one unique pocket estimation method despite pocket estimation uncertainties. In this paper, we propose ‘PockDrug-Server’ to predict pocket druggability, efficient on both (i) estimated pockets guided by the ligand proximity (extracted by proximity to a ligand from a holo protein structure) and (ii) estimated pockets based solely on protein structure information (based on amino atoms that form the surface of potential binding cavities). PockDrug-Server provides consistent druggability results using different pocket estimation methods. It is robust with respect to pocket boundary and estimation uncertainties, thus efficient using apo pockets that are challenging to estimate. It clearly distinguishes druggable from less druggable pockets using different estimation methods and outperformed recent druggability models for apo pockets. It can be carried out from one or a set of apo/holo proteins using different pocket estimation methods proposed by our web server or from any pocket previously estimated by the user. PockDrug-Server is publicly available at: http://pockdrug.rpbs.univ-paris-diderot.fr.

## INTRODUCTION

The ability of a protein to bind drug-like molecules, which are orally bioavailable, with a high affinity is often referred to as druggability, as first defined by Hopkins and Groom (1). Druggability assessment plays a key role in the first step of drug discovery project, lead identification or optimization phase that represents ∼60% of failure rate (2). Consequently, the computational evaluation of target druggability, prior to the investment of resources, has become crucial for the clinical progression of compounds. Many computational approaches have been developed to predict target druggability before extensive time and money are investigated and to reduce the high failure rate. These computational prediction methods involve pocket estimation, i.e. identifying the atoms that form the binding pocket (see Pérot *et al.* (3) for a review of pocket estimation methods), which is a key issue as there is no consensus pocket estimation method. Estimation of the same binding site using various estimation methods may result in different estimated pockets (4). Presently, each of the existing druggability models (DrugPred (5), Desaphy's model (6), fpocket score (7), SiteMap (8), DoGSiteScorer (9)) and existing web servers (fpocket website (10), DoGSiteScorer website (11), DrugEBIlity web service (available on the url: https://www.ebi.ac.uk/chembl/drugebility), iDrug website (12) or PLIC website (13)) can compute pocket druggability. However these models and websites are attached to one particular pocket estimation method despite pocket estimation uncertainties and are not optimized to be efficient using various estimation methods. Current computational druggability models based on pocket estimation methods guided by the ligand position (extracted by proximity to a ligand) give good results such as DrugPred (5) or Desaphy's model (6). In contrast, druggability models based on pocket estimation methods fully independent of the ligand position (automatically predicted as the atoms that form the surface of potential binding cavities) perform less well but are extendable to crucial druggability prediction of apo pockets (i.e. fpocket score (7) and DoGSiteScorer (9)).

In this context, proposing drugabbility web server able to accurately predict holo but also apo pocket druggability regardless the pocket estimation method used is required for drug discovery. In this paper, we present PockDrug-Server based on druggability prediction model constructed to be efficient for different pocket estimations methods (4). Compared to the existing druggability models, accuracies are ∼5–10% point higher than the results obtained in previous studies (6,8) on the same apo set. PockDrug-Server is a pocket druggability prediction server robust with respect to pocket boundaries uncertainties. Indeed, druggability prediction can be carried out from a protein or a set of proteins, using default pocket estimation methods, (guided or not by the ligand position) or from any pocket previously estimated by the user.

### PockDrug druggability model

#### Protein datasets and pocket estimation methods

The first set used to construct PockDrug druggability model was the largest currently freely available dataset, i.e. the ‘NonRedundant dataset of Druggable and Less Druggable binding sites’ (NRDLD), proposed by Krasowski *et al.* (5). The NRDLD set contains 113 non-redundant complex proteins sharing a pairwise sequence identity of less than 60% and includes a large diversity of enzymes, such as oxidoreductases, ligases and hydrolases. It corresponds to 71 pockets classified as druggable (i.e. binding site that non-covalently binds small drug-like molecules, which are orally available and do not require administration as prodrugs) and 42 as less druggable, as defined by Krasowski *et al.* (5). Later, this NRDLD set was split into one training set and one independent test set, based on the identical division used by Krasowski *et al.* (5) and Desaphy *et al.* (6).

The second pocket set used to validate PockDrug model when extrapoling to apo pockets, was collected from the ‘Druggable Cavity Directory’ (DCD) database (http://fpocket.sourceforge.net/dcd), proposed by Schmidtke *et al.* (7). It includes 139 proteins in the apo form (later referred to as Apo139), all of which have an equivalent holo form; 132 were classified as druggable.

During PockDrug model construction, different pocket estimation methods were considered: (i) estimation method guided by the ligand position information corresponds to the extraction of protein atoms localized within two fixed distance thresholds of 4 and 5.5 Å from a binding ligand, respectively known as prox4 and prox5.5; (ii) the two considered pocket estimation methods, not guided by the ligand position, are fpocket (14) and DoGSite (15), recently used (16–19) and proposing their own druggability prediction methods: pocket druggability score (7) and DoGSiteScorer (9). NRDLD pocket set was estimated using these four different pocket estimation methods. Apo139 pocket set was estimated using two different pocket estimation methods not guided by the ligand position.

#### PockDrug model construction

**Figure 1.**
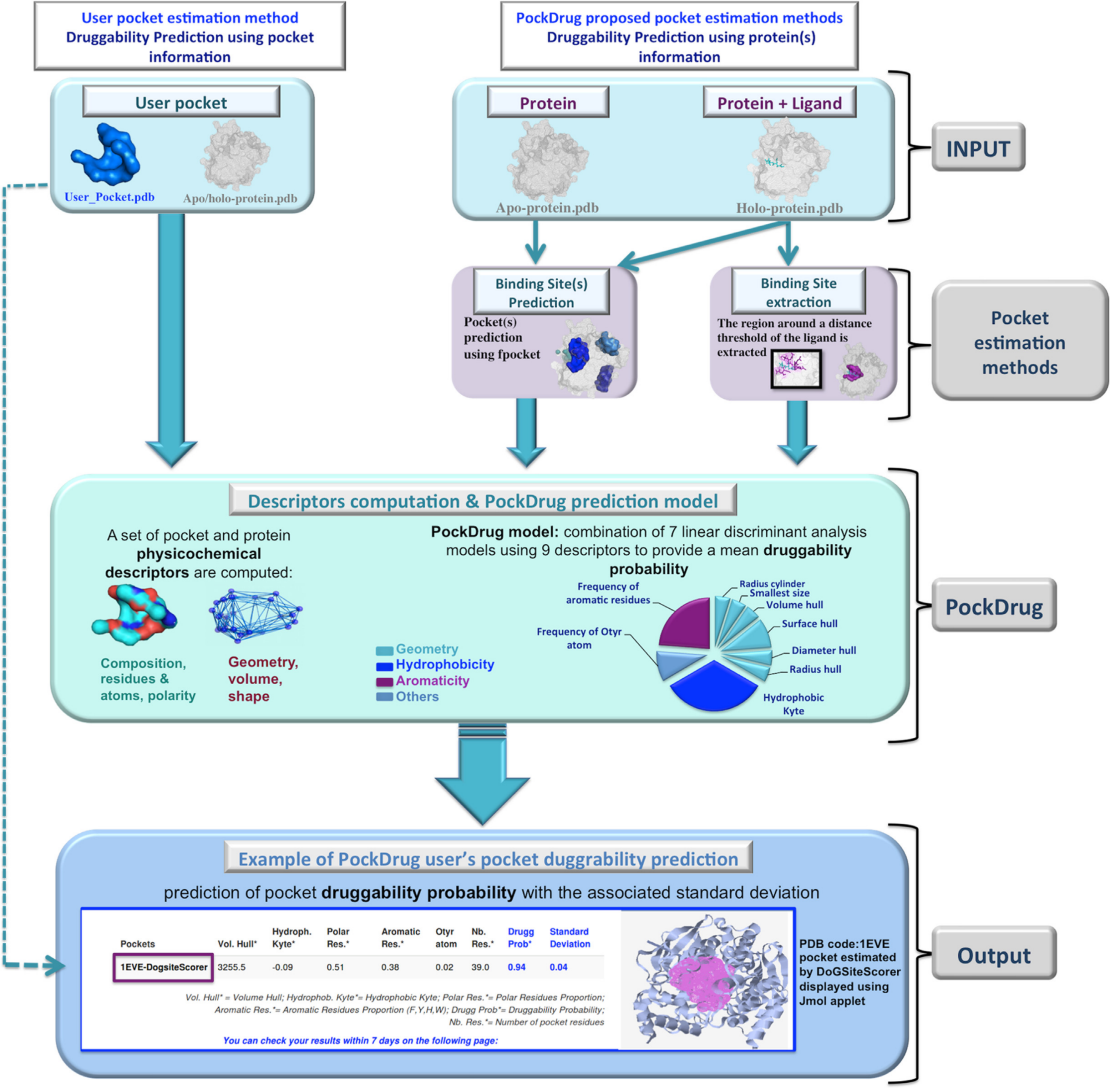
Workflow presenting the different steps of PockDrug-Server protocol. It is divided into four main parts, from top to bottom: input information (in clear blue), pocket estimation methods (in pink), PockDrug druggability prediction (in green) and the output display (in blue).

PockDrug model is an optimized druggability model develop by Borrel *et al.* (4) obtained from three statistical steps: (i) optimization of linear discriminant analysis models combining a selection of pocket descriptors from a pool of 52 geometrical and physicochemical descriptors by cross-validation using training part of NRDLD set estimated by fpocket, (ii) the selection of seven most stable and efficient models using NRDLD independent test set estimated using four different pocket estimation methods, (iii) the construction of one unique consensus druggability model: PockDrug from the best models. PockDrug model provides, as output, one druggability probability corresponding to the average of the probabilities of the seven best models and its associated standard deviation. PockDrug model is able to clearly distinguish a druggable pocket with an average druggability probability of 0.87 ± 0.15 from a less druggable pocket, with an average of 0.18 ± 0.15 using NRDLD set estimated by four estimation methods. PockDrug model selects descriptors that exhibit both the properties of being connected to druggability and robustness with respect to the pocket estimation uncertainties in order to efficiently predict the druggability of different estimated pockets. The seven best models included in PockDrug model are based on hydrophobic information combined with volume information, complemented by aromatic information or the pocket composition of hydroxyl group (see PockDrug part of Figure 1). For descriptors definition, see Supplementary Tables S3 and S4 of the Supplementary Data.

#### PockDrug model validation

The PockDrug model performances in terms of accuracy, sensitivity, specificity and Matthew's correlation coefficient (MCC) using NRDLD test set estimated by four different estimation methods (prox4, prox5.5, fpocket and DoGSite) have been evaluated in Borrel *et al.* study (4). Performance variability according to different pocket estimation methods illustrates the influence of the pocket estimations on the druggability performance. But interestingly, PockDrug model exhibits good performances with accuracies >83% (up to 94.6%) and MCC >0.650 (up to 0.885) whatever the pocket estimation methods are. This confirms PockDrug model ability to overcome the pocket estimation uncertainties to predict pocket druggability. Among druggability models based on pocket estimations not guided by the ligand position (fpocket score (7) and DoGSiteScorer (9)), PockDrug model outperformed by at least 10% points in terms of accuracy and 0.2 in terms of MCC using identically estimated test set.

Table 1 shows that PockDrug model applied to apo pocket set exhibited high performance with an average accuracy of 92.5% and a MCC of 0.48. PockDrug model increased MCC to more than 0.20 and the accuracy to more than 20% points compared to the two recent druggability prediction models, DoGSiteScorer and fpocket score applied to identically estimated apo pocket set. Taking into account pocket size for the pocket druggability prediction is crucial as pointed out by Gao and Skolnick (20). These authors excluded cavities having <10 residues from their pockets analysis. Hence, PockDrug-Server recommends a careful examination of pockets predicted as druggable and having <14 residues as these types of pockets correspond only to 10% of NRDLD and Perola druggable pockets (21) sets. For instance, pockets exhibiting >10 residues but <14 can correspond either to smallest druggable pockets or to decoy pockets (as defined by Hajduk *et al.* (22)).

**Table 1.** PockDrug, fpocket score and DoGSiteScorer druggability models performances in terms of accuracy, sensitivity, specificity and Matthew's correlation coefficient (MCC) using Apo139 pocket sets estimated using fpocket and DoGSite

Druggability models	PockDrug model		fpocket score	DoGSiteScorer
Pocket estimation methods	fpocket	DoGSite	fpocket	DoGSite
Accuracy	91.4	93.5	47.5	79.1
Sensitivity	92.4	94.7	44.7	78.8
Specificity	71.4	71.4	100	85.7
MCC	0.45	0.515	0.198	0.328

### The PockDrug-Server interface

#### Workflow

##### Input

The main function of PockDrug-Server is to predict pocket druggability. To do so, two types of query can be submitted:

(i) *Pocket structure*, which corresponds to a PDB format file listing the atom pocket coordinates. The corresponding protein PDB file is also required to compute pocket descriptors and their predicted druggability probability.
(ii) *Protein structure*, which corresponds to a PDB code, a PDB protein file or a file of PDB code list. For this type of query PockDrug-Server protocol is divided into two main steps:
  - Step 1: pocket(s) estimation using one or both different pocket estimation methods proposed by our web server;
  - Step 2: pocket druggability probability prediction. For each pocket, previously estimated in step 1, pocket druggability probability and standard deviation are provided by PockDrug model.

Protein query can be an apo or a holo protein. In the case of a holo-protein the ligand can be included in the protein file or uploaded apart so an optional field for ligand information is additionally proposed.

The user can also submit a single job for a set of apo or holo proteins in the form of a list of PDB codes. In this case the same previous protocol is applied so for each PDB code included in the list, pocket(s) is (are) estimated and its corresponding druggability probability is then predicted.

##### PockDrug-Server pocket estimation methods

When the submitted input corresponds to protein structure (Input case i) one or two proposed pocket estimation methods could be selected: prox or/and fpocket.

- *Prox* method, is based on ligand proximity information giving the user the possibility of choosing a threshold going from 4 to 12 Å (by step of 0.5 Å) in order to extract the protein atoms localized within the chosen distance of the ligand. Indeed, this threshold choice was recently shown to have a strong influence on the pocket descriptors (23) and it seems pertinent to give the user the opportunity to choose it. Two commonly used distance thresholds are recommended: 4 Å as used by Krasowski *et al.* (5), to enable the extraction of a well-defined pocket limited to short ligand interactions (as hydrogen bonds or ionic interactions) and 5.5 Å, to enable the identification of all significant contact points and a more complete environment of the binding site. This method is suitable for holo-proteins and threshold of 4 Å is chosen by default.
- *Fpocket* estimation method, not guided by the ligand information, is an automated geometry-based method based on the decomposition of a 3D protein into Voronoi polyhedrals. It extracts all the pockets from the apo- or holo- protein surface using spheres of varying diameters. Its advantages include calculation speed and satisfactory performance in terms of overlaying known binding sites with the predicted sites (7). This method is used by default since it is suitable for both apo- and holo- proteins.

##### Output: Pocket descriptors and druggability prediction

The output page may consist of one or two tab(s), varying accordingly to the choice of one or two estimation method(s): one result tab per selected estimation method. Relative to the input type, two result displays are possible:

(i) If the submitted query corresponds to a single pocket structure or a single protein entry (PDB code or PDB file) each tab is structured as following:
  (a) *Sortable table(s):* 1 or 2 tables can be provided for each query; one table corresponding to pockets having at least 14 residues, and if necessary, a second table displaying smaller pockets (<14 residues), in order to help the user to distinguish the decoys from pockets:
    (1) In table 1, corresponding pockets with more than 14 residues are large enough to bind a ligand and the druggability probability can be directly interpreted.
    (2) In table 2, pockets with a number of residues ranged between 10 and 13, displayed in grey, have to be examined carefully. Cavities with <10 residues, too small to be considered as pockets, are indicated in grey, italic and highlighted using ‘°’ sign.
    These tables provide for each pocket:
      - 6 out of the 17 pocket descriptors available in PockDrug-Server (see PockDrug Model construction section and Supplementary Table S3). As pocket estimation method affects directly the descriptors values, the descriptor averages with associated standard deviations computed on NRDLD set estimated using three different estimations (prox4, prox5.5 and fpocket) are given as reference (Supplementary Table S4) to facilitate the user analysis of the pocket descriptor values.
      - The average druggability probability and its associated standard deviation that indicates the druggability probability confidence on the seven best models included in PockDrug. For a probability greater than 0.5, pockets are considered as druggable. In the case where several pockets are considered, the table can be ordered in ascending or descending order of druggability probability to facilitate the identification of druggable pockets.
  (b) *Pocket visualization using the Jmol web browser applet* (24) pocket(s) and protein structures can be visualized and manipulated on the server through jmol applet. All computed results: pockets structures, eleven descriptors and druggability scores can be downloaded.
  (c) *Compressed file containing all the results* can be downloaded using the download button. Only when the pockets are estimated using both fpocket and prox (for all distance threshold), overlapping scores between two pocket estimations are also computed and provided to the user through the compressed result file in order to allow pocket estimation comparisons and correspondence between two estimation methods. See section of pocket comparison in the supplementary data for the definition of overlapping scores. A bookmark button saving the link on which the user can follow the evolution of his job, access and download it within 7 days.
(ii) If the submitted query corresponds to a list of PDB codes, each tab corresponds to a sortable table showing:
  - Protein PDB code: giving access to the detailed result page as it is described previously in this paragraph (case i)
  - Number of estimated pockets (for each method)
  - Number of druggable pockets (druggability probability greater than 0.5)
  - The highest druggability probability and its standard deviation

#### Computation time

Once the job is submitted, all the input data is checked and the computation begins on our server. A holding page, giving the link on which the user can check the progress of his job is displayed while awaiting the end of calculations. Computation time is relatively short; it mainly depends on the protein and pocket size and the choice of estimation methods as well. Generally, the computation takes approximately a minute to a medium-sized protein or a pocket entry. Calculation time can be longer for a dataset (file of PDB code list). During the submission, the user can optionally provide his email address to be notified once the job is done and receive results. This option is recommended in the case of a PDB list.

In order to test the server and to reduce computational time delays, the whole RCSB Protein Data Bank (25) was downloaded, all pocket estimations using two proposed estimation methods (prox4 and fpocket) and pocket descriptor computations were performed. Resulting associated druggability probabilities of pockets estimated were pre-computed and stored on our servers. Therefore, displaying results tends to be relatively fast. Calculations for the other prox method thresholds are being generated. A monthly automatic update of our database is implemented by adding new PDB entries that are recently included in the PDB. If one user query is a PDB code not pre-included in our database, the results will be added to our pool.

#### Web server implementation

The entire web server has been implemented using Python, HTML, CSS and Javascript. Jmol Applet has been used for the protein 3D structure visualization. The descriptor computation scripts have been written in python and bash while the PockDrug LDA model has been developed using R. External softwares have been also used: fpocket (14), RADI, available in website http://petitjeanmichel.free.fr/itoweb.petitjean.freeware.html, NACCESS V2.1.1, available in the website http://www.bioinf.manchester.ac.uk/naccess/ and Open Babel (26).

A comprehensive help section is available on the website to help the user understand each of the steps involved and infer the output of the results produced in the workflow.

#### Acetylcholinesterase, an example of target with several pockets predicted as druggable

**Figure 2.**
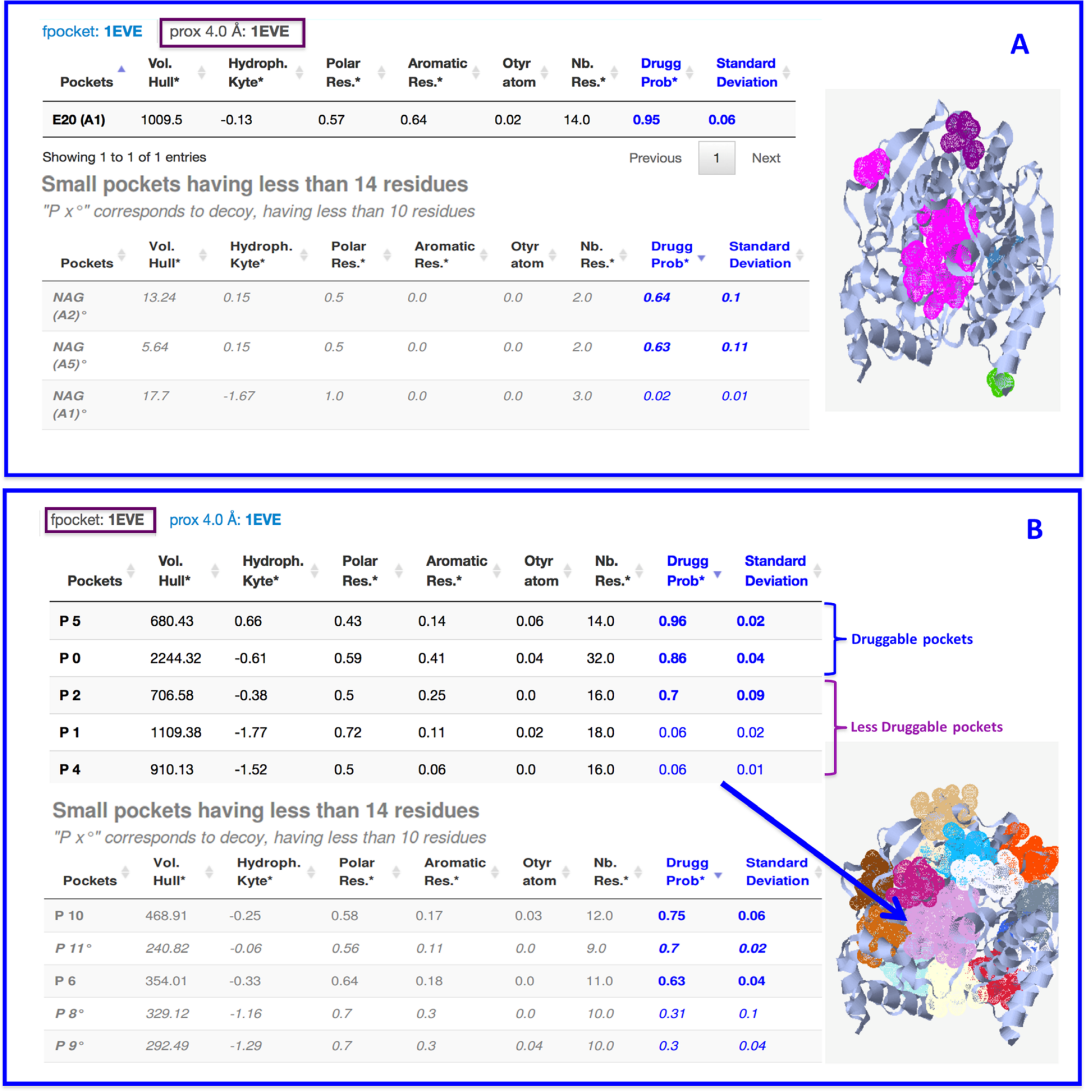
Example of PockDrug-Server output using acetylcholinesterase protein structure (1EVE). (**A**) The results display of pocket estimated using prox4 estimation method sorted by descending order of druggability probability; (**B**) the result display of pocket estimated using fpocket estimation method sorted by descending order of druggability probability.

Here, we present an example of the acetylcholinesterase (AChE) protein. AChE Irreversible inhibitors may have various serious effects some of which can be fatal. In contrast reversible inhibitors, occupying also the esteratic site, are used to treat a range of central nervous system diseases. Several cholinesterase inhibitors are currently either being used for symptomatic treatment of Alzheimer's disease or are in advanced clinical trials. We choose the AChE holo-protein complexed with the anti-alzheimer drug E20 marketed as Ariceptand (PDB code 1EVE) (27). This protein consisting of 534 residues, was used for recent computational investigations (28,29). AChe, denoted as 1eve for the rest of this study, was not included in the training set used to build PockDrug model. The E20 binding pocket is tested by PockDrug-Server. A first query consists on the using of an external pocket estimation method (DoGSite, input case i), (see results in output part of the Figure 1). PockDrug-Server returns an average druggability probability of 0.98 (±0.01). When the same pocket is estimated and directly predicted by DoGSiteScorer (9), an ambiguous druggability score of 0.56 is assigned for this pocket, ranking it as a ‘potentially/difficult druggable pocket’. PockDrug-Server result is in a good agreement with the literature (30) and the annotation of DCD database confirming that E20 binding site corresponds to a druggable pocket. 1EVE pockets are also estimated using PockDrug-Server default estimation methods (prox4 and fpocket) and are displayed in Figure 2. Corresponding druggability prediction was performed in ∼50 s. Overlapping scores (see Supplementary Data, Supplementary Tables S1 and S2) show that estimated pocket noted P0 by fpocket corresponds to the pocket binding, noted E20 by prox4 (Figure 2A). PockDrug-Server results in consistent high average druggability probabilities of 0.95 (±0.06) when estimated by prox4 (Figure 2A), of 0.97(± 0.02) when estimated by prox5.5 and of 0.86 (±0.04) when estimated by fpocket (Figure 2B). Whatever the pocket estimation method is, PockDrug druggability predictions are relatively similar confirming that in this case PockDrug model is able to overcome the pocket estimation uncertainties.

Interestingly, in addition to pocket binding E20, out of 16 estimated pockets using fpocket estimation method PockDrug-Server predicted five other pockets as druggable. These five pockets correspond to high druggability probabilities (more than 0.63) and could be of interest in the case of AChE, which is an important target to treat alzheimer disease and other central nervous system diseases. So combining different pocket estimation methods, especially those that do not need any *prior* ligand position information, allows the identification of new potential druggable pockets.

N-Acetyl-D-Glucosamine (NAG), a natural co-enzyme, is also present in 1EVE PDB, as a ligand, binding four different pockets on the surface of this protein. As shown in Figure 2, the pocket binding the NAG2 analysed by PockDrug-Server, using prox4 is predicted as ‘may be druggable’ (0.64 ± 0.1) and confirmed as druggable pocket with a mean druggability probability of 0.7 (±0.02) when estimated by fpocket. This case of figure shows the importance to have different pocket estimation methods and a robust model to compare pockets. It proves also that identifying new druggable pockets can be beneficial to propose other drugs candidates.

## DISCUSSION

PockDrug-Server proposes druggability prediction from pocket estimated using different pocket estimation methods. Compared to the existing models, accuracies are ∼5–10% points higher than the results obtained in previous studies on the same apo set.

On one hand, the originality of PockDrug-Server is that it provides the average druggability probability with its corresponding standard deviation that gives an idea about the predictions variation and thus we can be confident that the result will occur.

On the other hand, different methods of pocket estimation that have been used to construct PockDrug model enable us to provide, to our knowledge, the unique free web server proposing a druggability prediction that overcomes the limits and inaccuracies of pocket estimations and is efficient with different pocket estimation methods.

In addition, the opportunity given by our web server to directly submit a pocket of interest, estimated based on the user expertise in the domain is of major importance.

The fact that PockDrug-Server accepts as input any structure in the PDB format, the user can estimate and predict pockets druggability not only for X-Ray or nuclear magnetic resonance structures but also for models obtained by homology (apo form) or by docking (holo form).

Many services are combined and proposed by PockDrug-Server: (i) predicting pocket druggability probability for any estimated pocket regardless to what pocket estimation method is used, (ii) proposing two pocket estimation method(s) (iii) in the case where the pocket is estimated by proximity to the ligand, a range of distance threshold can be chosen by the user (default 4 or 5.5 Å), (iv) proposing to the user to use his own estimation method based on his knowledge of the protein and pockets or in the case of pockets difficult to estimate (v) submitting a protein set is also possible to facilitate the research—which is not the case with other druggability web servers and (vi) finally all the results can be visualized online and can be also downloaded with some supplementary data (overlapping scores, descriptors reference average) that help users understand and analyse the results of their job.

## CONCLUSION

PockDrug-Server is part of an important initiative of our team study to provide an online tool for computer-aided drug discovery. PockDrug-Server could be, for instance, useful for druggability assessment to evaluate whether a compound development project is worth starting after an X-ray structure is available. It is also beneficial for ranking/prioritizing the pockets of an orphan protein to choose one suitable pocket for compound development in structure-based drug design or finding a druggable secondary pocket as well as for identifying a target in a disease-modifying pathway to compare druggability predictions and select which protein is most pertinent to study in a set of proteins. The future aim will be constructing a pipeline to allow the user to predict pocket druggability probabilities, to profile drug-like molecules for each druggable pocket or to predict any interaction that it could have with targets different than its primary target in an attempt to predict any side effects.

## SUPPLEMENTARY DATA

Supplementary Data are available at NAR Online.

SUPPLEMENTARY DATA
