# Supplementary material for: PockDrug-Server: a new web server for predicting pocket druggability on holo and apo proteins
Source: Nucleic Acids Res. 2015 May 8;43(Web Server issue):W436–42. doi: 10.1093/nar/gkv462 (PMC4489252; doi:10.1093/nar/gkv462)
Supplement: SUPPLEMENTARY DATA [file supp_43_W1_W436__index.html]

PockDrug-Server: a new web server for predicting pocket druggability on holo and apo proteins — PockDrug-Server: a new web server for predicting pocket druggability on holo and apo proteins — SUPPLEMENTARY DATA 

# PockDrug-Server: a new web server for predicting pocket druggability on holo and apo proteins

## SUPPLEMENTARY DATA

- SUPPLEMENTARY DATA
