# Supplementary material for: PockDrug-Server: a new web server for predicting pocket druggability on holo and apo proteins
Source: Nucleic Acids Res. 2015 May 8;43(Web Server issue):W436–42. doi: 10.1093/nar/gkv462 (PMC4489252; doi:10.1093/nar/gkv462)
Supplement: SUPPLEMENTARY DATA [file supp_gkv462_nar-00582-web-b-2015-File004.pdf]

POCKET COMPARISON

If two default pocket estimation methods (prox and fpocket) are tested, overlapping scores between these two pocket estimations are computed and included in the downloaded compressed result file, in order to compare pocket estimates. The overlap between two estimated pockets was quantified using two scores:

- Score of Overlap (SO) indicates the overlap between the two pocket estimates, i.e., pocket1 and pocket2, as follows:

$$SO = \frac{Ncommon}{Npocket1 + Npocket2 - Ncommon}$$

where Npocket1 and Npocket2 are the number of atoms in pocket1 and pocket2, respectively, and Ncommon is the number of atoms common to pocket1 and pocket2. SO yields values between 0 and 100%. An SO value of 100% indicates maximum overlap between the pair of estimated pockets used.

- Relative Overlap (RO) and Mutual overlap (MO) was defined by Schmidtke et al. (1) and indicates the overlap in terms of the exposed area between the two estimated pockets for the same binding site:

$$RO = \frac{SApocket1 \cap SApocket2}{SApocket1}$$

$$MO = \frac{SApocket1 \cap SApocket2}{SApocket2}$$

where SApocket1 and SApocket2 are the solvent-accessible areas of pocket1 and pocket2, respectively, computed using NACCESS software. An RO and MO values closer to 1 indicates all exposed area in pocket1 are included in pocket2. RO and MO are complementary, relative to the pocket 1 for the RO and for the pocket 2 for the MO

Table S1. Table giving the Mutual Overlapping (MO) between pockets estimated using prox4 and fpocket for the protein structure 1EVE (only when at least one pocket estimated by prox4 overlaps at least one pocket estimated using fpocket).

		prox4 ←				
		E20	NAG_A_1	NAG_A_2	NAG_A_4	NAG_A_5
**fpocket** ↓	P 9	0.0	0.11	0.0	0.0	0.0
	P 5	0.0	0.0	0.0	0.0	0.33
	P 11	0.0	0.0	0.78	0.0	0.0
	P 0	0.96	0.0	0.0	0.0	0.0

Table S2. Table giving the Score of Overlap (SO) between pockets estimated using prox4 and fpocket for the protein structure 1EVE (only when at least one pocket estimated by prox4 overlaps at least one pocket estimated using fpocket).

		prox4 ←				
		E20	NAG_A_1	NAG_A_2	NAG_A_4	NAG_A_5
**fpocket** ↓	P 9	0.0	0.03	0.0	0.0	0.0
	P 5	0.0	0.0	0.0	0.0	0.27
	P 11	0.0	0.0	0.19	0.0	0.0
	P 0	0.38	0.0	0.0	0.0	0.0

POCKET DESCRIPTORS

Table S3. Definition of the seventeen descriptors available in the PockDrug-server output

Descriptors	Description	References
	**Hydrophobicity descriptors**	
Hydrophobic kyte	Hydrophobicity based properties of residues	Kyte et al. 1982 (2)
Hydrophobic residues	Proportion of hydrophobic residues in pocket (C, G, A, T, V, L, I, M, F, W, Y, H, K)	
	**Polarity descriptors**	
Polar residues	Frequency of polar residues in pocket (C, D, E, H, K, N, Q, R, S, T, W, Y)	
	**Aromatic descriptors**	
Aromatic residues	Frequency of aromatic residues in pocket (F, Y, H, W)	
	**Physicochemical descriptors**	
Aliphatic residues	Frequency of positive residues in pocket (I, L, V)	
Otyr atom	Frequency of Otyr atoms in pocket	Milletti et al. 2010 (3)
Ne2 atom	Frequency of NE2 atoms in pocket	Milletti et al. 2010 (3)
Nlys atom	Frequency of Nlys atoms in pocket	Milletti et al. 2010 (3)
Ntrp atom	Frequency of Ntrp atoms in pocket	Milletti et al. 2010 (3)
Ooh atom	Frequency of Ooh atoms in pocket	Milletti et al. 2010 (3)
Nd1 atom	Frequency of ND1 atoms in pocket	Milletti et al. 2010 (3)
	**Geometric descriptors**	
Surface hull	Surface of convex hull ($Å^2$)	RADI software
Diameter hull	Longest distance in the convex hull (Å)	RADI software
Volume hull	Volume of convex hull ($Å^3$)	RADI software
Smallest size	Distance separating the two closest slabs enclosing the hull (Å)	RADI software
Radius cylinder	Radius of the smallest height cylinder enclosing the hull (Å)	RADI software
Nb RES	Number of pocket residues	

Table S4. The Nine PockDrug model descriptor averages with associated standard deviations computed on NRDLD set estimated using three different estimations (prox4, prox 5.5 and fpocket) to be used as reference. In addition the Number of pocket residues is also presented

Descriptors	prox4	prox5.5	fpocket
Hydrophobic kyte	-0.43 ± 1.17	-0.33 ± 1.11	-0.31 ± 0.99
Otyr atom	0.015 ± 0.02	0.009 ± 0.01	0.012 ± 0.015
Aromatic residues	0.216 ± 0.16	0.19 ± 0.13	0.17 ± 0.1
Radius hull (Å)	8.52 ± 1.78	10.19 ± 1.76	11.71 ± 3.84
Surface hull ($Å^2$)	448.71 ± 162.64	724.571 ± 204.46	939.20 ± 582.82
Diameter hull (Å)	16.82 ± 3.64	20.16 ± 3.57	23.09 ± 7.65
Volume hull ($Å^3$)	752.0 ± 408.45	1614.52 ± 686.23	2532.92 ± 2472.48
Smallest size (Å)	8.38 ± 1.68	11.36 ± 1.667	12.044 ± 3.44
Radius cylinder (Å)	8.307 ± 1.79	9.98 ± 1.77	11.45 ± 3.82
Nb RES	14.67 ± 5.53	21.88 ± 7.26	30.90 ± 18.22

1. Schmidtke,P. and Barril,X. (2010) Understanding and predicting druggability. A high-throughput method for detection of drug binding sites. *J. Med. Chem.*, **53**, 5858–67.

2. Kyte,J. and Doolittle,R.F. (1982) A simple method for displaying the hydropathic character of a protein. *J. Mol. Biol.*, **157**, 105–32.

3. Milletti,F. and Vulpetti,A. (2010) Predicting polypharmacology by binding site similarity: from kinases to the protein universe. *J. Chem. Inf. Model.*, **50**, 1418–31.
